# Supplementary material for: Homogeneous Polymerization of Kraft Lignin Using an Alkaliphilic Multi-Copper Oxidase (Bilirubin Oxidase) in a Borate Buffer
Source: Polymers (Basel). 2025 Mar 14;17(6):779. doi: 10.3390/polym17060779 (PMC11944997; doi:10.3390/polym17060779)
Supplement: Supplementary file 1 [file polymers-17-00779-s001.zip › polymers-3524667-supplementary.pdf]

polymers

MDPI

Article

Homogeneous Polymerization of Kraft Lignin Using an Alkaliphilic Multi-Copper Oxidase (Bilirubin Oxidase) in a Borate Buffer

Lou Delugeau [1], Aurèle Camy [1], Léna Alembik [1], Philippe Poulin [2], Sébastien Gounel [2], Nicolas Mano [2], Frédéric Peruch [1,*] and Stéphane Grelier [1,*]

Supporting information

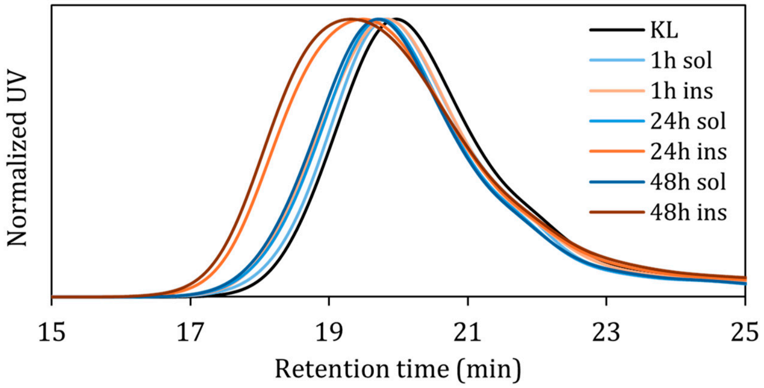

Figure S1: SEC chromatograms (UV detection) of Kraft Lignin and fractions from control solubilizations at different reaction times. “1h sol” refers to the soluble fraction obtained after 1h solubilization. Same for other fractions with a time variation and the presence of soluble/insoluble fractions.

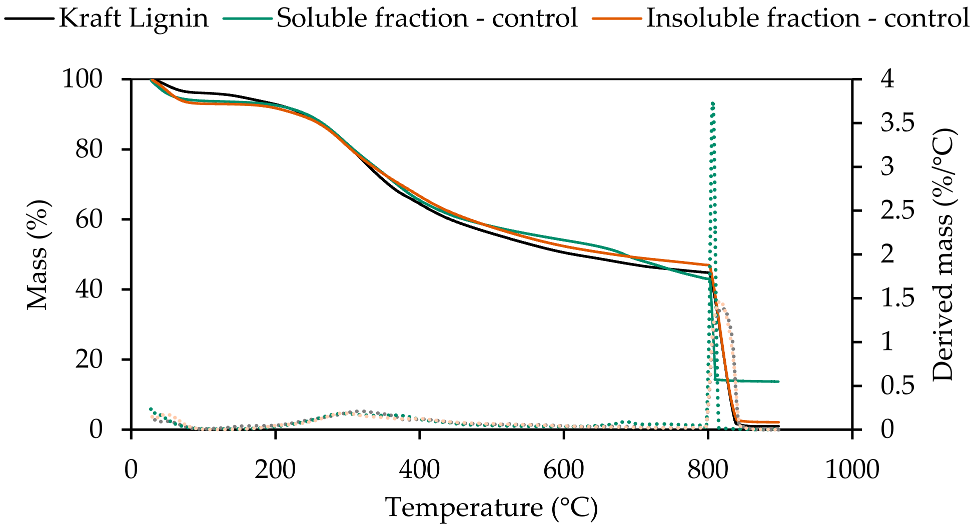

Figure S2: Thermogravimetric analyses (TGA) of Kraft lignin (black) and the soluble and insoluble fractions obtained from the control solubilization in a borate buffer at pH 10. Solid lines represent mass loss, while dashed lines correspond to derivative mass curves.

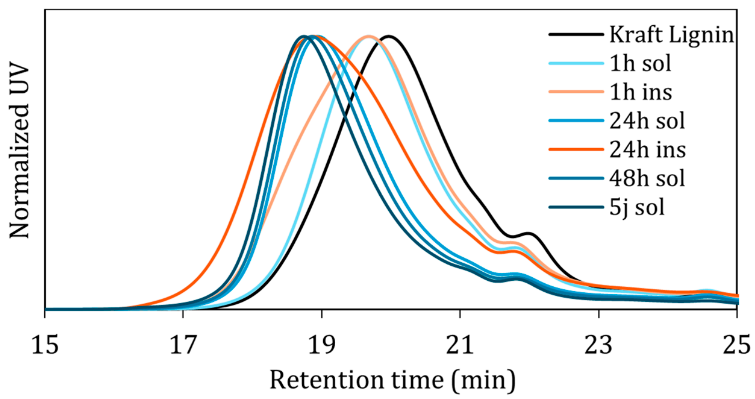

Figure S3: SEC chromatograms (UV detection) of Kraft Lignin and fractions from BOD enzymatic treatments at different reaction times. "1h sol" refers to the soluble fraction obtained after 1h of enzymatic treatment. Same for other fractions with a time variation and the presence of soluble/insoluble fractions.

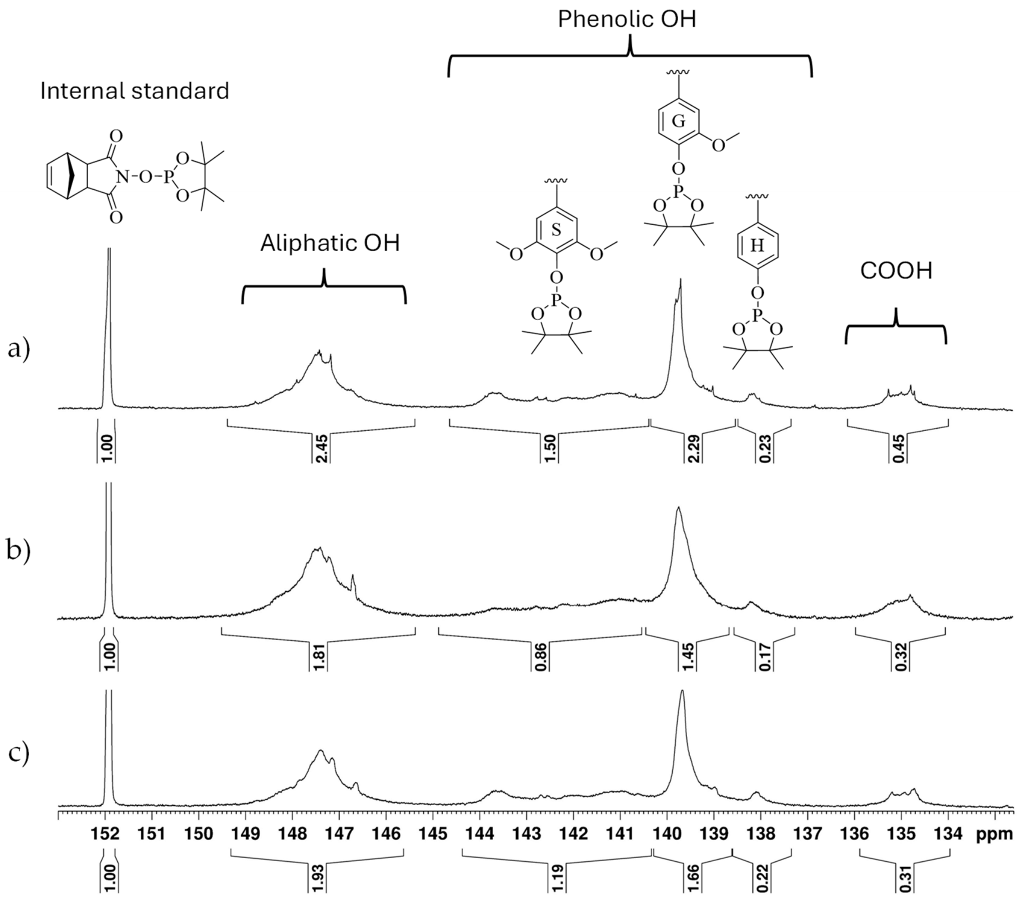

Figure S4: Quantitative ^{31}P NMR in $CDCl_3$ of (a) Kraft lignin; (b) control-soluble fraction; (c) "Sol_BOD".

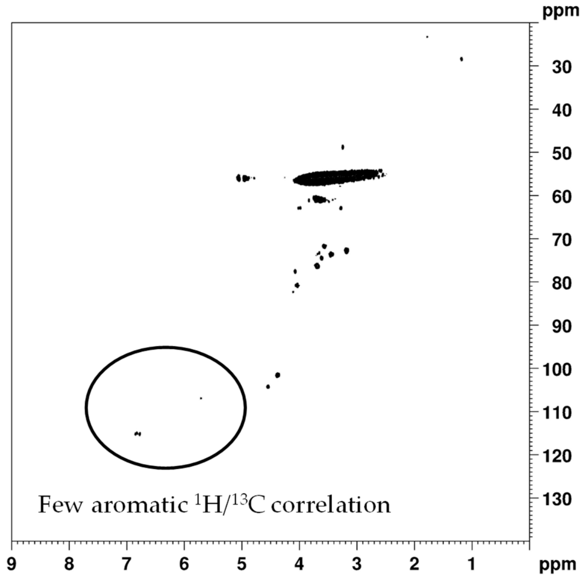

Figure S5. ^{1}H-^{13}C HSQC-2D NMR spectra in D_2O of the soluble fraction from the lignin Kraft BOD treatment
